# See[ing] elegance in sensory biology: an interview with Piali Sengupta

**DOI:** 10.1242/dmm.050321

**Published:** 2023-06-12

**Authors:** Piali Sengupta

**Affiliations:** Department of Biology, Brandeis University, Waltham, MA 02454, USA


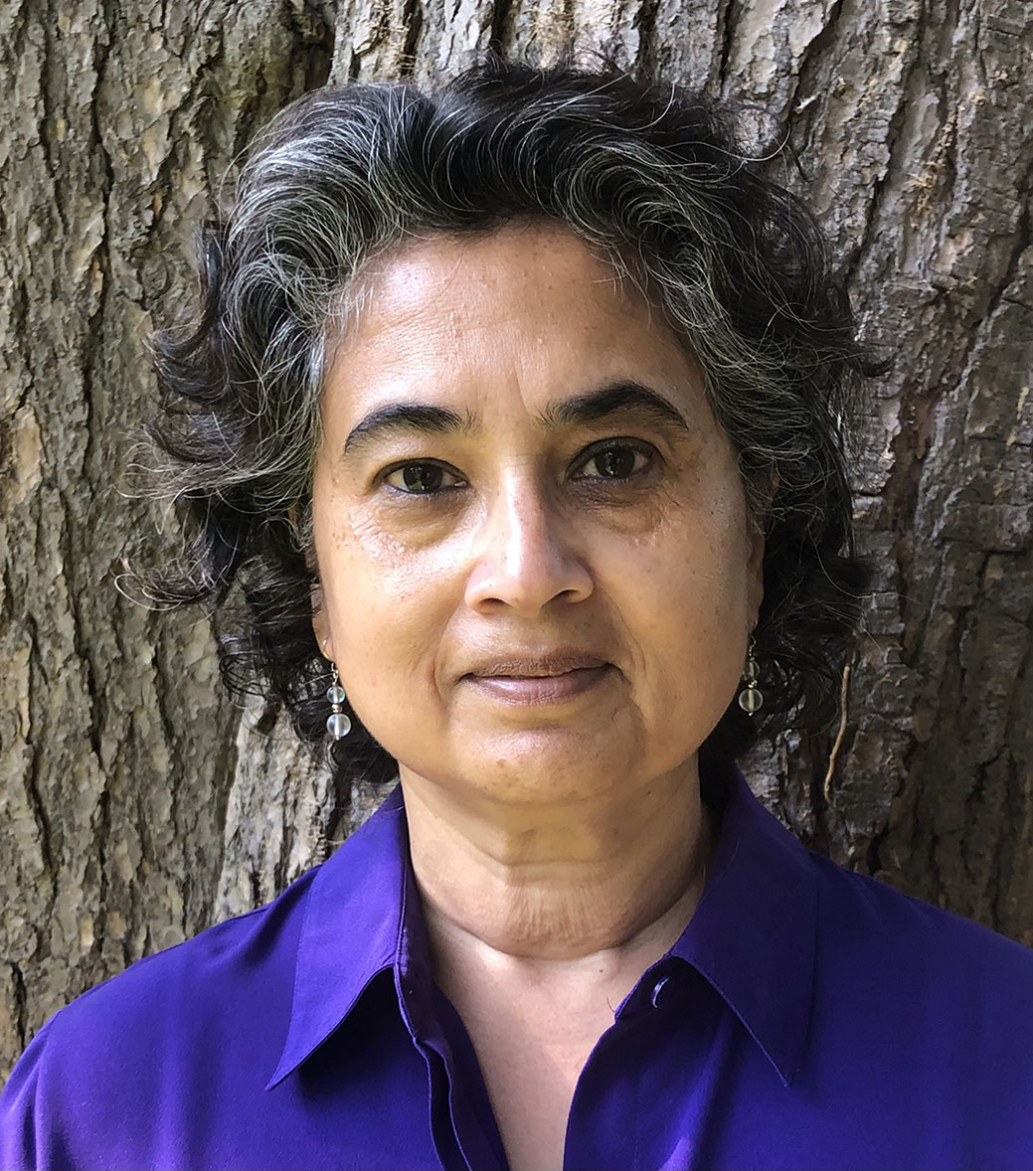


Professor Piali Sengupta harnesses the power of *Caenorhabditis elegans* to enrich our understanding of sensory biology. She approaches fundamental biological questions by dissecting highly conserved – and unique – sensory mechanisms in this experimental organism*.* As a core member of the ‘worm community’, she advocates for fundamental research, but also highlights how robust findings in this elegant system can be translated to inform human physiology and disease.

Piali did her PhD in Brent Cochran's laboratory at the Massachusetts Institute of Technology and pursued a postdoc in Cori Bargmann's laboratory, then at the University of California San Francisco. After starting her own laboratory at Brandeis University, she has held many scientific positions, including editorial board member on multiple journals, member of grant review panels, and officer in scientific organisations, such as the Genetics Society of America. In addition to supervising her research laboratory, she devotes significant time and energy to outreach and mentoring programmes. In this interview, Piali discusses the power of the worm in studying sensory mechanisms, and highlights the support and strength of the *C. elegans* research community.

**Can you outline the main advantages of using *C. elegans* to study sensory mechanisms?**

One of the main things to consider when studying sensory mechanisms in experimental organisms is that they must actually display the behaviour of interest robustly and reproducibly, but this behaviour must also be plastic – meaning that it can be influenced by the animal's past experience, the environment and its internal state. Worms meet all these criteria. They have a huge number of complex behaviours that can be studied really well in the lab, which is one major advantage. Their small size also helps enormously. Several years ago, Cori Bargmann's lab developed tiny microfluidic devices for monitoring worm olfactory behaviour at high resolution, and, because the worms are so small, you can do this in a high-throughput manner. This allows you to, for example, figure out what specific aspect of their behaviour is being affected by a gene mutation and you can assess worm behaviour in real time – not just collect an endpoint measurement. People are now studying behaviour in detail in other animals, such as mice, using other techniques, but it is super easy to do in worms.

Of course, the complete nervous system of *C. elegans* has been mapped. You can kill most neurons in the worm and they will normally survive in the lab, which helps you to assess the role of specific neurons in behaviour. The worms are also transparent, so you can measure intracellular calcium dynamics in the live, behaving animal without any major invasive techniques. Forward genetic screens are also very easy because the majority of the worm population in the lab are hermaphrodites. If you have a mutant hermaphrodite, you just put it on a plate, and it reproduces. This is a huge advantage because experiments can progress really quickly. And now, of course, CRISPR works incredibly well in worms.

I've been working with worms for over 30 years at this point, and every day I am amazed by the fact that we can go from identifying a mutant in a single gene and then easily see the effect this mutation has on specific neurons, the neuronal circuit and, finally, the behaviour. It never ceases to amaze me, and, for all of these reasons, worms are a really remarkable system for looking at sensory behaviours and responses.

**You provide a lot of support to the *C. elegans* research community, such as organising meetings and mentoring. Why is this such an important endeavour?**

The worm community is quite unique in how collaborative and interactive it is. This was established by the people who actually started the field in Cambridge at the MRC Laboratory of Molecular Biology. That culture has pervaded the community and has persisted in it, which is quite remarkable over so many years. I joke that I'll email somebody for a reagent, and it will arrive the next day by express mail, which is not actually greatly exaggerated, because it does literally happen. Worm researchers are willing to share unpublished data and other resources, as people tend to collaborate, not compete. I've been very lucky to be a part of this community, so I've tried to give back to it and to the people in my lab, and I've tried to foster the same kind of openness, because the community has done so much for me. I feel like I want to pay it forward.

People in this community are also very willing to mentor you. I'm fairly senior in my field now, but I still ask people to read my papers or grants and they will come back with very, very honest (and sometimes brutal) feedback, which is really useful. So, when people send me papers and grants to read and give advice on, I try to do it because people do it for me. It's something that's not true for every community, and I really appreciate it. There is also a *C. elegans* mentor match programme that's run by the WormBoard. People sign up to be mentors, and then the board matches mentors with mentees. They are obviously not in the same institution, so this essentially allows junior researchers to network and form connections, which is so important.

“[…] although studying conserved mechanisms is important, studying divergence is equally important. It tells you a lot about how evolution has found multiple solutions to a problem […]”

**Have you encountered any challenges when communicating *C. elegans* research to a broader research community?**

I think this is a challenge that everybody who works on an experimental organism that doesn't have a backbone experiences. When we write grants, we often receive the comment, ‘How does this apply to humans?’, or ‘This is not translatable’. But I think things are changing a bit. The advent of CRISPR has opened people's eyes to the fact that you can use all kinds of cool organisms for research and that it doesn't have to be restricted to mice or rats. I have pretty strong opinions about this because I think that, although studying conserved mechanisms is important, studying divergence is equally important. It tells you a lot about how evolution has found multiple solutions to a problem, and it tells you a lot about the underlying mechanisms of this. If the same physiological response or outcome can be achieved in ten different ways, that's really interesting and important to learn about.

It is also very short sighted to assume that certain mechanisms are worm, or *Saccharomyces* or *Drosophila* specific, because there's a chance that three decades later you find that the process or molecule is super important to humans as well. microRNAs are an amazing example of that, as they were thought to be worm specific for a really long time, and now we all know how important microRNAs are throughout phyla. I always say to people that we're biologists, so why shouldn't we be interested in finding out how all organisms work? The translational angle is important, but I think basic research is equally important and should be funded, and this is hopefully starting to be more appreciated now.

“[…] I think the sensory neurons are doing a lot more than just sensing and responding to information, and it'll be very interesting to see what the mechanisms of this are […]”

**With the 2022 Nobel Prize being awarded for work investigating receptors for touch and temperature, this is an exciting time for sensory biology. In your opinion, what have been the most interesting recent discoveries in this area of research?**

The discovery of Piezo has been fascinating as it is a molecule that is involved in mechanosensation in many different contexts from plants to humans. But there are a couple of other discoveries that I think are very interesting, although a little more esoteric.

First, sensory neurons are often thought of more as passive transducers of sensory information, so they're sensing whatever environmental stimulus is present and transmitting it to higher brain centres, which are then doing all the integration and a lot of higher-order computations. However, in worms, we've known for a long time that sensory neurons do a lot more. We've always thought that this might be worm specific because they have a very shallow sensory network architecture: it just goes from sensory neurons, to interneurons, to motor neurons, then to muscles and to behaviour, and it doesn't have all the layers of the brain like you have in more complex animals. But it turns out that, even in mammals, sensory neurons can perform very complex computations. This has now been shown in mouse olfactory neurons (Tsukahara et al., 2021; von der Weid et al., 2015) and also in fly sensory neurons (Sung et al., 2023). In these studies, long-term sensory experience or internal state changed the transcriptional properties of the sensory neurons themselves, leading to experience-dependent changes in behaviour. This was originally thought to occur due to computations in higher brain centres, but has now been shown to also happen in the periphery. And so, I think the sensory neurons are doing a lot more than just sensing and responding to information, and it'll be very interesting to see what the mechanisms of this are, and also to see if this is applicable broadly across multiple types of sensory neurons.

The second field that has progressed in the past few years is the concept of interoception. We know a lot about how we sense external information, like light, touch, smell and temperature, but we don't know as much about how we sense information from our internal organs through interoception, such as airway and stomach stretch. Our brain needs to get information from the liver, the kidneys, the heart and the lungs, and a lot of that is mediated by sensory neurons in the vagus nerve that sense chemical and mechanical information. But how is it doing that? What are the different types of stimuli that these vagal neurons are sensing? How are these neurons gathering all this information, integrating it and sending it to the brain? I think this is a new area in sensory biology that people are starting to study using modern experimental approaches. It's obviously not as applicable to worms, but it's a super interesting field.

**What discovery or research project have you found most exciting during your own career?**

One of the things that I've consistently been fascinated by in worms is their ability to sense temperature. Worms have been referred to as champion thermal sensors, as they can sense temperature differences of as little as 0.01°C and change their behaviours accordingly. They can remember the temperature at which they were grown, and if you put them on a temperature gradient, they'll go towards that remembered temperature. Also, if you expose them to a different temperature, they can reset their memory, and then their preference for temperature will change as well. Most amazingly, they do all of this complex behaviour pretty much using just a single pair of thermosensory neurons. This has been really fascinating, and we keep finding more and more interesting things about how this neuron pair functions. It has a beautiful structure, and it has complex mechanisms for encoding temperature information and then driving the subsequent behaviours. There have been a tonne of surprises when studying this, and the community has been very important because we've collaborated with many people to learn how these neurons function.

This research area is something we've been consistently working on for quite a long time, and it's been frustrating sometimes too. I started my lab with a lot of work on chemosensation, but about half of my lab now works on temperature sensation, and I think the temperature sensation work has been absolutely fascinating. There are lots more questions to be asked here, which keep me both interested and challenged at the same time.

“It is very easy in this field to feel justified using experimental organisms to study these human diseases, because there's direct translation, which is quite remarkable.”

**How can fundamental research into these mechanisms help us better understand human disorders, such as ciliopathies?**

One of the most amazing things about cilia is how incredibly conserved the core mechanisms are for their formation and function. A lot of the initial work on cilia was done in organisms like *Chlamydomonas*, *Tetrahymena* and *Paramecium*, and, of course, worms. Then, decades later, it was found that almost every cell in the human body is ciliated, and the cilia are formed by the same mechanisms across the phyla. Furthermore, mutations in the conserved genes involved in these mechanisms can result in human developmental disorders, known as ciliopathies. In worms, it's just the sensory neurons that are ciliated, unlike in humans, in whom almost all cells are. So, we specifically investigate cilia in sensory neurons in worms, but even then, a lot of the genes we found to be involved in cilia formation and function are conserved. We recently published a paper in which we identified a gene in a forward genetic screen that has also been implicated in Joubert syndrome in humans (Maurya and Sengupta, 2021).

This is a subject area where people who work on cilia in organisms like protists and worms never have to justify their work because so many major findings were actually made in these organisms. There are, of course, species-specific and cell-specific cilia mechanisms as well, but, overall, the conservation is really quite remarkable. Therefore, this field is really unusual, as people keep up with literature in pretty much every organism because it is clear that you can learn so much by studying the biology across species. At a cilia meeting, there will be people who work on cilia in mammals, and they'll know more about the worm cilia field than I will, and people switch between model organisms in their research too. There's also a lot of connection with human patients. There are researchers who take mutations in cilia genes that have been identified in ciliopathy patients and engineer those mutations in worms to see what mechanistic effects those mutations are having and to get a better idea of what mechanisms are being affected in the human patients. Furthermore, at some of the cilia meetings, we will have patients with ciliopathies attending, which has been very powerful as well. It is very easy in this field to feel justified using experimental organisms to study these human diseases, because there's direct translation, which is quite remarkable.

“We can't lock ourselves up in a lab and just do our work. We're part of a community and of society, and we have to engage everybody in it.”

**You are also involved in a lot of public outreach activities. Why is accurate and engaging science communication so important?**

Yesterday, I spent a lot of time preparing a syllabus to teach biology in a local correctional facility. It's part of a programme that provides incarcerated students an opportunity to obtain a degree, and is part of some of the outreach efforts I participate in. I also take part in a programme at a local science museum where we run science programmes for children. We organise hands-on activities for the children and their parents, and we talk to them about the science we're doing in our own labs. It's fun to see how interested they are, and it's a way of engaging more kids in STEM, as opposed to scaring them off.

Science obviously doesn't exist in a vacuum, and we're funded by federal funds, which is paid for by taxpayers. So, I think it's important to communicate back to the public what we're doing with this money and why it is important. I think general education about science and scientific principles is really important for everyday life, regardless of your profession. This helps people to make sense of what probability means in genetic testing, for example, or to understand the caveats of scientific findings in the news, or to understand all the misconceptions around mRNA vaccines. It's also important for politicians who make the funding decisions to understand what basic research is and how important it is, as well as to understand that translating basic research to a drug doesn't happen overnight. It takes a really long time, with a lot of industry and biopharma work, but the foundation of the work is from basic research, often in an academic lab. It's important for people who are in positions of power to understand the process itself.

I'm also often asked, ‘Why do you work on worms? Why don't you do something more important, like work on a cure for cancer?’ So, then I try to explain to them why working on worms is important. This is something that should be part of everyone's general education and it's one of the reasons I do this. We can't lock ourselves up in a lab and just do our work. We're part of a community and of society, and we have to engage everybody in it. So, we try, but I wish I had more time to do it.

**What do you enjoy doing outside of work?**

I work a lot, but one thing I really like doing is travelling to remote places with very bad weather. My whole family likes to travel, so every year we choose a place for our vacation, and we usually end up somewhere where it is snowing in summer, we will be covered in mud, there will be nobody around, and there is gorgeous scenery. That's my favourite type of place to go to.

I also read a tonne. I read a lot of fiction – all kinds of genres – and I'll go through one to two books a week. I also like music – the louder the better! I've been trying for a while to teach myself how to play the drums, and I'm really bad at it. I'm trying, but I don't seem to have any talent for it, so I do it in the privacy of my own house.
